# Professional perspectives on providing recovery-oriented services in Taiwan: a qualitative study

**DOI:** 10.1186/s12888-021-03152-y

**Published:** 2021-03-16

**Authors:** Yen-Ching Chang, Ling-Hui Chang, Su-Ting Hsu, Meng-Wen Huang

**Affiliations:** 1 Department of Occupational Therapy, College of Medicine, National Cheng Kung University, Tainan, Taiwan; 2 Institute of Allied Health Science, College of Medicine, National Cheng Kung University, Tainan, Taiwan; 3 Kaohsiung Municipal Kai-Syuan Psychiatric Hospital, Kaohsiung, Taiwan; 4 Department of Rehabilitation Science, Jenteh Junior College of Medicine, Nursing and Management, Miaoli, Taiwan

**Keywords:** Recovery-oriented services, Professionals, Attitudes

## Abstract

**Background:**

The experiences of professionals in well-established recovery-oriented programs are valuable for professionals in similar practice settings. This study explored professionals’ experiences with providing recovery-oriented services in community psychiatric rehabilitation organizations.

**Methods:**

Semi-structured interviews were conducted with 14 professionals from five recovery-oriented psychiatric rehabilitation organizations in Taiwan. The interviews were recorded and transcribed verbatim. Thematic analysis was used for the qualitative data analysis.

**Results:**

The analyses documented three main themes with 13 subthemes. Recovery-oriented service implementation included seven subthemes: Enabling clients to set their own goals and make decisions, using a strengths-based approach, establishing partnerships with clients, improving individuals’ self-acceptance, encouraging community participation, seeking family, peer, and organizational support, and building team collaboration. Problems with implementing recovery-oriented services included limited policy and organizational support, a lack of understanding of recovery among professionals, stigma, clients’ lack of motivation or self-confidence in their own ability to achieve recovery, and passive or overprotective family members. Strategies to resolve implementation problems included policy changes and organizational support, improving the recovery competence and confidence of professionals, and family and public education.

**Conclusions:**

To date, this is the first known study examining the perspectives of mental health professionals who have experience implementing recovery-oriented services in Asia. The participants identified family collaboration, anti-stigma efforts, and changes in policy and attitudes as critical to successful implementation and delivery of recovery-oriented services.

**Supplementary Information:**

The online version contains supplementary material available at 10.1186/s12888-021-03152-y.

## Background

Recovery is regarded as a guiding vision of the mental health service system [1], shifting the focus of services from symptom maintenance to personal growth and choices. The origin of the recovery concept can be traced back to the era of deinstitutionalization in the US [1]. Because of limited community services, advocates began to strive for the civil rights of people with mental illness as well as for alternative mental health services [2, 3]. The idea of recovery emerged and provided an alternative service framework. Although there is yet to be a consensus on the definition of recovery-oriented services, such services mainly focus on helping people with mental illness set and achieve their own recovery goals and improve their degree of life satisfaction [4–6].

People with mental illness can recover with various support sources, and mental health professionals can be part of the support system. Transforming the traditional medical model into recovery-oriented services requires the development of a new set of values, service goals, and practice implementations on the part of mental health professionals [6, 7]. This includes establishing positive therapeutic relationships with people with mental illness, encouraging them to express their feelings, giving them hope, supporting their decisions, and encouraging them to take responsibility for their own recovery [4, 8, 9]. Such service models not only focus on internal changes in individuals, but also on the determinants of environmental and social health [10]. Therefore, recovery-oriented services involve a wide range of areas, including medication, personal care, goal setting, and social interaction, all of which require collaboration among various mental health professions [4, 11].

Two systematic reviews [11, 12] have documented the perspectives of mental health professionals on recovery-oriented services and the obstacles they encounter when implementing such services, such as limited resources and support from organizations, a lack of understanding of recovery, and clients’ poor motivation to set and achieve recovery goals [4, 10, 13]. However, few studies have explored how recovery services are implemented in non-Western societies, where individualistic ideas, such as personal growth and empowerment, are only beginning to gain attention, and social expectations regarding family caregiving responsibilities is high. In such sociocultural contexts, mental health professionals may experience distinct challenges related to implementing recovery-oriented services that are different from those of their Western peers.

Taiwan has begun to implement recovery-oriented services [14]. The recovery concept was introduced into the accreditation standard of psychiatric rehabilitation organizations in Taiwan in 2017 [15]. However, many mental health professionals, despite having been educated about recovery, still struggle to successfully implement these services. Experiences and examples shared by professionals in well-established recovery-oriented programs can provide valuable, practical insights for professionals in similar practice settings. Therefore, the aim of this study was to explore the experiences of professionals from well-established recovery-oriented organizations in Taiwan, including how they implement recovery-oriented services, the problems they face, and potential strategies for overcoming these implementation problems.

## Methods

### Research context

Currently, there are 226 community psychiatric rehabilitation organizations in Taiwan funded by National Health Insurance, including 71 day programs and 159 housing programs [16]. Day programs provided structured activities, such as leisure and vocational activities, for clients, most of whom are living with their families. Housing programs provide similar daytime activities, as well as independent living skills training, to residents.

### Participants

Purposive sampling was used to recruit professionals from well-established recovery-oriented organizations. We invited three accreditation committee members of psychiatric rehabilitation organizations, who have visited many organizations in Taiwan, to recommend candidates. Five organizations were selected, including 3 day programs and two housing programs, to achieve a fair representation of the type of organization and establish regional diversity. The number of staff members in these organizations ranged from four to thirteen. We contacted the heads of the organizations and asked them to recommend two to four experienced staff members to participate in individual semi-structured interviews.

### Data collection

This study was approved by the lead author’s university’s Institutional Review Board. Data collection was conducted from August 2017 to February 2019. Trained interviewers (YC and MH) interviewed the participants, who were given the interview guide beforehand in the meeting room used for their organizations. The interview guide was developed based on previous literature [10, 11] and the research objectives of this study. The questions included how the recovery-oriented services were implemented, what problems were encountered when providing such services, and what strategies they suggest to resolve these problems. Each interview lasted one to one and half hours, and was recorded and transcribed verbatim. Data collection continued until no new information was obtained since this indicated data saturation.

### Data analysis

Thematic analysis is a method used to analyze and report themes emerging from data. It can be conducted within an essentialist framework to explore the experiences of participants [17], which fits the needs of the current study. Atlas.ti 8.0 software (Scientific Software Development, Berlin, Germany) was used for data coding. We analyzed the data in a deductive manner; i.e., the analysis was driven by our research interest. Two researchers familiarized themselves with the data, read the transcripts line by line, and generated the initial codes. For example, *“In the beginning, clients are encouraged to make small decisions, such as choosing which courses they want to attend, or what time they can arrive at the organization. Gradually, we encourage them to try other things.”* was coded as “Empowerment” and “Encourage to try things” to represent how the recovery-oriented service was implemented. Next, the two researchers reviewed, combined, and organized the codes and developed preliminary themes. For example, “Empowerment”, “Client-centered”, and “Taking responsibilities” were merged into the theme of “Enabling clients to set their own goals and make decisions”. These codes and themes were reviewed by the research team until a consensus was reached. Finally, clear definitions and names were generated for each theme, and the structure of the themes was confirmed.

Triangulation and member checks were used to ensure data credibility [18, 19]. We recruited five organizations located in different areas of Taiwan and interviewed professionals from different professions to enrich the breadth and depth of the data. In addition, we sent each participant the transcript and interpretation summary to confirm that our interpretation was consistent with their experiences with implementing recovery-oriented services.

## Results

We interviewed 14 professionals from five community psychiatric rehabilitation organizations in Taiwan. The interviewed professionals ranged in age from 23 to 53 years old, with a mean age of 41. Their professions included psychiatry, nursing, occupational therapy, social work, and case management. Years of experience providing mental health services ranged from 1.5 to 28 years (see Table 1).

**Table 1:** Participant characteristics

	*N* = 14
Gender, n(%)	
Female	11((79%)
Education, n(%)	
Bachelor’s degree	7 (50%)
Master’s degree or above	7 (50%)
Profession, n(%)	
Psychiatrist	2 (14%)
Nurse	3 (21%)
Occupational therapist	4 (29%)
Social worker	3 (21%)
Case manager	2 (14%)
Age (years), mean ± SD	41.07 ± 8.85
Work experience in mental health services (years), mean ± SD	14.46 ± 8.62

The analyses documented three main themes with 13 subthemes: Recovery-oriented service implementation, problems with implementing recovery-oriented services, and strategies to resolve implementation problems (see Table 2).

**Table 2:** Overview of themes and subthemes

Themes/Subthemes (numbers of quotations)	Definitions of subthemes
1. Recovery-oriented services implementation (214)	
Enabling clients to set their own goals and make decisions (46)	Professionals create opportunities for clients to select, plan, and take actions in their lives based on their needs and expectations. Clients learn and grow from the decision-making process.
Using a strengths-based approach (18)	Professionals acknowledge clients’ strengths and promote clients’ use of their unique capabilities, which can help them develop hope for their future.
Establishing partnerships with clients (31)	Professionals interact and discuss matters with clients as if they are friends.
Improving individuals’ self-acceptance (13)	Professionals help clients gain a better understanding of mental illness and the concept of recovery in order to accept their own situation and redefine themselves.
Encouraging community participation (28)	Professionals encourage clients to interact with the community and gain more life experiences.
Seeking family, peer, and organizational support (44)	Professionals seek support and resources from various sources, including families, peers, and organizations.
Building team collaboration (34)	Professionals reach a consensus on recovery-oriented services and exhibit consistent attitudes toward clients.
2. Problems with implementing recovery-oriented services (159)	
Limited policy and organizational support (46)	Policies and organizations have insufficient support for recovery-oriented services.
A lack of understanding of recovery among professionals (20)	Professionals have a limited understanding of and practice with recovery-oriented services.
Stigma (30)	Stigma on mental illness impedes professionals from promoting recovery-oriented services.
Clients’ lack of motivation or self-confidence in their own ability to achieve recovery (36)	Clients’ low motivation or self-confidence hinder the implementation of recovery-oriented services.
Passive or overprotective family members (27)	Families with unsupported attitudes restrain the implementation of recovery-oriented services.
3. Strategies to resolve implementation problems (82)	
Policy changes and organizational support (27)	Support from policy and organization level boosts the implementation of recovery-oriented services.
Improving the recovery competence and confidence of professionals (32)	Strengthening professionals’ recovery competence and confidence is beneficial for implementing recovery-oriented services.
Family and public education (23)	Education to improve attitudes of families and the public is critical for implementing recovery-oriented services.

### Theme 1: recovery-oriented service implementation

How professionals implemented recovery-oriented services included the professionals’ attitudes toward their clients and the methods they use to deliver services. The content below provides an example of how one participant (Occupational Therapist D) implemented recovery-oriented services.

“This client has a master’s degree, and he had worked previously as an engineer. When he searched for jobs, he looked for positions like engineers and administrative planning. We told him, ‘If you want to try different jobs, go ahead. We will support you.’ After he went to an interview, we followed up his situation. Then, he shared with us his interview or job search process. He found out that, for example, in one of his interviews, the employer stated, ‘I asked A, and you answered B or C to me. What you answered was not what we wanted.’ Later when he went to another interview, the employer said, ‘You said you had a master’s degree, but according to the way you speak and react, I feel like you don’t have such a high degree of education at all! Did you lie to us?’ Well, the reality of society forced the client to directly face the pressure of real life … However, he still didn’t believe it and kept trying different jobs. He has worked as a security guard, a worker in a beverage shop, and a project assistant … However, he did not have successful work experiences. Also, he found out that he seems to forget things easily. For example, when he worked in the beverage shop, he easily forgot the steps required to make a drink. He had to ask others how to do it, and kept asking over and over again... The boss was very angry and asked, ‘Have you studied hard? Did you remember [the procedure]?’ The client wondered to himself, ‘I have listened carefully. Why can’t I remember?’ He also started to wonder why he couldn’t do things as well as others … It took him several years to gradually accept his own situation and reexamine his goals. Therefore, I think that recovery-oriented services are time-consuming, labor-consuming … quite labor-consuming … We need to build a team consensus. When a client comes, we have to be consistent with what we say to the client and make sure our attitudes are also consistent. However, it helps the individual become aware of the real situation and triggers change. This client gradually realized that he should not compare himself with others, but rather should be looking at himself. If he makes progress, it is worthy of joy and recognition.”

This example showed how the professional provided support for her clients, and how her client learned and grew during the process. Seven subthemes emerged from the data: Enabling clients to set their own goals and make decisions, using a strengths-based approach, establishing partnerships with clients, improving individuals’ self-acceptance, encouraging community participation, seeking family, peer, and organizational support, and building team collaboration.

### Enabling clients to set their own goals and make decisions

The participants’ frequently mentioned using a client-centered approach to help clients set their recovery goals and plans based on their individual needs and expectations. In addition, the participants created opportunities for clients to select, plan, and take actions in their lives, such as activities of daily living, travel, and employment. For example: “In the beginning, clients are encouraged to make small decisions, such as choosing which courses they want to attend, or what time they can arrive at the organization. Gradually, we encourage them to try other things” (Social Worker I). Clients can learn from their own first-hand experiences, where a failed outcome helps them make different decisions in the future.

### Using a strengths-based approach

Many participants used a strengths-based approach when interacting with clients. They understood their clients’ strengths and promoted the clients’ use of their own unique capabilities. One stated, “When he was put into that situation [where students with special needs were guided by him to paint the wall], he was very excited to be called “teacher” since he had never had similar opportunities or experiences” (Occupational Therapist N). These experiences enabled people with mental illness to have hope, to see their own growth and strengths, and incentivized them to strive towards their recovery goals.

### Establishing partnerships with clients

Some participants mentioned that they routinely established partnerships with their clients. This meant that they interacted and discussed matters with their clients as if they were friends: “We always hope that we are connecting with them as friends, rather than from a top-down position. We want to maintain an approach where we engage in mutual discussions with them on how they should act, rather than simply asking them to do what we are requesting them to do” (Social Worker L).

### Improving individuals’ self-acceptance

Some participants helped clients gain a better understanding of mental illness and the concept of recovery in order to accept their own situation and redefine themselves. Open-ended questions were used to encourage discussions, providing clients with opportunities to reflect on their life journey and face their inner emotions. “We discussed this incident [e.g., frustration at work] in front of the whole class, asking them if they have had similar experiences or emotions. They started to understand how to face their own life situations and themselves. We also encouraged them to accept their mistakes … what they did in the past” (Occupational Therapist E).

### Encouraging community participation

Since many Taiwanese people with mental illness have limited opportunities to participate in various activities, the participants encouraged their clients to engage with others outside of the organization and interact with the community, which allowed them to have more life experiences. One participant stated, “We connected with some community resources. Our drum team went to teach children or the elderly to play drums. Gradually, some organizations invited us to perform. Our clients also painted the school walls together with the schoolchildren. At the same time, this activity also helped schoolchildren understand what people with mental illness are like” (Social Worker I).

### Seeking family, peer, and organizational support

Seeking support from various sources, e.g., family members, peers, and organizations, was critical for implementing recovery-oriented services. The participants noted that the connection between family and people with mental illness is tightly knit and mutually influential. One said, “Chinese culture is different from Western culture. When we make decisions, we usually consider what our family will think. We grow up this way...” (Psychiatrist A). Hence, the implementation of recovery-oriented services requires continuous communication with family members. One participant stated, “We continue to provide the content of these rehabilitation plans each month to the family. In terms of recovery, we are not simply working with the client but also with the entire family” (Social Worker L).

Peers function as an important source of social support. The participants encourage people with mental illness to help their peers and share their recovery experiences, with the hope that it will boost their confidence in their own recovery. For example: “We had the opportunity to have two clients share their recovery experiences in public. Hearing their peers sharing successful experiences had a positive impact on some of our clients” (Psychiatrist M).

When facing limited resources, mental health professionals work with other organizations to pool resources. One participant mentioned, “We used to organize sports competitions on our own. Later, we approached other organizations to co-organize the event with us. By taking the first step, we are demonstrating to others that we already have experience. There will then be organizations that decide to follow our lead and work with us” (Nurse K).

### Building team collaboration

Some participants stated that training from experienced professionals on how to interact with clients, as well as group discussions and supervised meetings helped team members build consensus on recovery-oriented services. Consistent attitudes toward clients among different professionals improved team collaboration and were beneficial for implementing such services. For example: “We discuss the client condition together and then treat the client consistently...We emphasize that our attitudes are consistent, and we do not intervene too much” (Case Manager H).

### Theme 2: problems with implementing recovery-oriented services

Problems with implementing recovery-oriented services included five subthemes: Limited policy and organizational support, a lack of understanding of recovery among professionals, stigma, clients’ lack of motivation or self-confidence in their own ability to achieve recovery, and passive or overprotective family members.

### Limited policy and organizational support

Our results showed that there was insufficient support for recovery-oriented services from both policies and organizations, such as unmatched reimbursement regulations and limited personnel. The participants felt that insurance reimbursement was inadequate to support the activities needed to implement recovery-oriented services. For example, when a client was discharged from an organization when he/she got a job, the insurance reimbursement was discontinued even though the professionals were still providing ongoing support to facilitate the client’s successful transition to paid employment. Such situations created a dilemma for the organizations and made them reluctant to push clients out. In addition, limited personnel and organizational resources made it difficult for participants to spend time with their clients and provide varied activities for clients. One said, “The level of policy support for [psychiatric] rehabilitation treatment is insufficient for us to develop recovery activities. We have to expend additional time seeking external resources” (Nurse K).

### A lack of understanding of recovery among professionals

Some participants suggested that relatively little attention is being paid to community psychiatry and the concept of recovery in professional education. One stated, “Our professional educational system is very much focused on pathology, but the outcomes of pathology will over-medicalize recovery for people with mental illness” (Psychiatrist M). The participants also mentioned that the concept of recovery is abstract, and practical opportunities to gain experience are insufficient, resulting in some professionals in their organizations not understanding how to deliver recovery-oriented services even if they were aware of the concept of recovery.

In addition, a paternalistic perspective continues to permeate mental health service delivery. Hence, participants frequently have to struggle between insisting on their preferred treatment for clients vs. respecting clients’ choices (e.g. relinquishing the power over treatment decisions). One participant mentioned, “By returning the sense of control to clients, we cease to be their guides. Instead, we become mere companions. I think this is extremely difficult for healthcare professionals” (Occupational Therapist E).

Participants also found that some staff in their organizations were skeptical about recovery. They are afraid of potential risks and believe that people with mental illness are not capable of taking responsibility. One said, “Some colleagues asked in a doubtful manner, ‘Do you really intend to let the client take over such major decision-making? Making these decisions may cause greater danger or risks to them. Are you going to be responsible for these?’” (Occupational Therapist D).

### Stigma

Stigma not only has a negative impact on the recovery of people with mental illness, but also impedes the ability of professionals to promote recovery-oriented services. For instance, some participants described how the construction of new community settings has resulted in issues such as protests and resistance from local residents. Stigma is also prevalent in vocational rehabilitation. One participant has done job matching for clients and stated, “We are well aware of how significant social stigma is for our friends with mental illness. It is quite common for employers to specify they do not want to hire people with mental illness” (Occupational Therapist F).

### Clients’ lack of motivation or self-confidence in their own ability to achieve recovery

Many participants commented that people with mental illness are relatively passive and are used to relying on their family as well as societal resources. This presents a dilemma for the participants between encouraging clients to strive towards meeting their potential or respecting their desire to be content with their current situation. One mentioned, “He can use the [governmental disability] subsidies to have the lifestyle he desires. He does not have any other material wants. As long as he and his family get along, he thinks life is good. You can only go along with his ideas. To us, he does not have the motivation to change anything” (Occupational Therapist F).

The participants also discovered that some people with mental illness lack self-confidence about their own recovery or are afraid of the challenges they may encounter when making changes. One said, “He has experienced hurt in the past. He requires a long time to get over such experiences, so once he has been in a very good state, he may feel that being in the current state is safer since the pressure is overwhelming, and there is no need to experience that again” (Social Worker I).

### Passive or overprotective family members

The participants frequently mentioned that client recovery is negatively affected when families do not actively take part in or support their relatives’ recovery journey, or families are unable to work collaboratively with their relatives. One stated, “Many families are not able to collaborate with our rehabilitation plans. We work with the client for his goals here, but once he gets home, he may be led into to a completely different lifestyle. So, he remains stuck in the same spot. If family members can collaborate with us, his progress will be much faster” (Social Worker L).

Furthermore, the participants also stated that some families are overprotective of their relatives with mental illness or are afraid to make changes. This results in people with mental illness being unable to practice independent learning and decision-making. Some participants said, “It is rather a pity that the family could not bear to see him suffer [from failures or getting rejected, etc.], so he quit the job. Our training thus could not be sustained. We very much need the support of families” (Social Worker I). “Maybe something in the past causes the family to be scared. They are afraid that if they let the client go out and try something different, it is possible the client comes back [with a relapse] and makes a mess again” (Nurse C).

### Theme 3: strategies to resolve implementation problems

Three subthemes emerged when discussing strategies to resolve implementation problems: Policy changes and organizational support, improving the recovery competence and confidence of professionals, and family and public education.

### Policy changes and organizational support

Specific policies enacted by the government to encourage or request organizations to implement recovery-oriented services may be one way of boosting organizational willingness to implement such services. For instance: “If an organization can help certain clients secure jobs, the government can increase reimbursement. This will provide a strong motivation for organizations.” (Occupational Therapist E). “If policies request the client management teams to hire peer specialists, then there will be comprehensive changes” (Psychiatrist M). The government could assist by consolidating the support networks among organizations to advance resource utilization. “This year, we are also making efforts to expand the networks for mutual resources. These networks can be established much more efficiently if they are undertaken by governmental departments” (Social Worker L).

Support from the organizations’ management teams was also found to be an essential element for implementing and sustaining recovery-oriented services. Some participants stated that if they can demonstrate to the management teams the effectiveness of such services, as well as the greater benefits that can be brought about with successful recovery, the management teams will support recovery-oriented services. One said, “Our own professionalism must also be sufficient to convince the boss that these expenses are within an acceptable range and also lead to good effects. Thus, the better you are at it, the better the organizational reputation will be over time. This is a bonus for the organization; perhaps this may lead to more applicants joining the organization” (Nurse G).

### Improving the recovery competence and confidence of professionals

The participants indicated that medical education and on-the-job training requires greater incorporation of and focus on recovery concepts. One stated, “If we wish to ensure a consistent direction for all staff members, there could be more professional courses related to this topic that we could attend” (Case Manager J). In addition, on-the-job training is also needed to strengthen the skills needed to implement recovery-oriented services, such as providing specific clinical guidelines or inviting experienced professionals to offer practice-based courses on the implementation of recovery-oriented services. One participant stated, “I think it is important for them [professionals] to come observe how it is done, attempt on their own, and then come back for a discussion. This is a process that requires back and forth reiterations, as there are many organizations who may know what to do when they observe us, but find they are unable to work with these methods on their home ground. This is because the implementation of recovery concepts depends on the environment and individuals.” (Occupational Therapist E).

In addition to educational and operational support, the participants also noted that successful service implementation can help change the opinions of professionals and increase their confidence when providing recovery-oriented services. One participant noted, “Actually, we have seen many successful cases, so we do not necessarily need to debate with them (other staff). It is challenging to change mindsets. We can first gather those who are willing to implement these services together, demonstrate effectiveness, and then help people understand that this is how it should be. Thereafter, we will gradually be able to convince others who disagree to think otherwise” (Occupational Therapist E). Another participant said, “When you receive gratitude or feedback from clients or their families, or when they have successfully found jobs, you will feel that your efforts have not gone to waste because there are indeed achievements” (Nurse G).

### Family and public education

Many participants commented that family members’ attitudes have a great impact on people with mental illness. One participant said that “Families can be educated. Do not be afraid of speaking to them. You need to communicate with them constantly and share examples with them. Tell them explicitly what the client has done and the positive outcome that has ensued” (Case Manager H).

Furthermore, the participants believe when the public has a better understanding of mental illness, this will reduce stigma and misunderstandings about people with mental illness and provide more opportunities in the community for them. People with mental illness need to be encouraged to speak up and learn to advocate for themselves. Public education on recovery also is needed. For example, “We can’t just simply practice within our organizations behind closed doors. We may need to bring our clients out and even help them learn to self-advocate. For example, we can have some performance opportunities. I think these are forms of self-advocacy” (Occupational Therapist N).

## Discussion

To the best of our knowledge, this is the first qualitative study in Asia of the perspectives of mental health professionals who have experience with implementing recovery-oriented services. This study documented their experiences when implementing these services, as well as implementation problems and strategies for resolving these problems.

Le Boutillier and colleagues reviewed 30 recovery-oriented service guide documents (all from Western countries) and developed a conceptual framework consisting of four domains: Promoting citizenship, organizational commitment, supporting personally defined recovery, and working relationship [20]. Although the categorizations are different, the implementation of recovery-oriented services described by Taiwanese professionals has many similarities. Probably because Taiwanese professionals learned the concept of recovery from the literature, a similar operational framework was used, such as a client-centered and strengths-based approach, as well as collaboration among people with mental illness and various support systems intended to successfully deliver recovery-oriented services. The above traits do not indicate any obvious cultural differences and can be considered to be universal features of such services.

In addition, many participants in this study mentioned the role of family dynamics in implementing recovery-oriented services. This phenomenon is understandable considering the culture context of Taiwan. Song found that family support is important for the recovery of Taiwanese people with mental illness [21], and scholars in Hong Kong also specifically list “family participation” in their defined recovery elements [22]. In Chinese culture, the connection between the family and the individual is close and interdependent. When a family member is sick, the family is expected to be responsible for the welfare of the sick person. Family members frequently influence the decision-making of their relative. The autonomy of the sick person become restricted [23–25], which can undermine self-determination. Our results showed that even though the participants were aware of the importance of the family and wanted to involve the family in their clients’ recovery process, collaboration with families can be problematic. Family members with passive or overprotective attitudes often prefer maintaining the status quo as opposed to risking possible frustration and negative consequences. One participant stated that professionals need to not only work with the client, but also with the family. Moving the family from being an obstacle to a catalyzer for client recovery is a major challenge for professionals within Chinese culture.

Based on our results, stigma has a great impact on the ability to implement recovery-oriented services. Stigma is a major obstacle to social integration of people with mental illness, and a stumbling block for their recovery [4, 26, 27]. Studies have shown that stigmatization of mental illness, such as associating mental illness with public danger, is more prevalent in Asian societies than in Western societies [28, 29]. Specifically, Confucianism, which emphasizes relationships and filial piety, makes suffering from mental illness extremely shameful in a Chinese context. People with mental illness whose behaviors deviate from Confucian values are stigmatized. Also, both those with mental illness and their family members are negatively impacted by stigma [30, 31]. Part of the reason family members tend to maintain the status quo may be related to stigma because making changes may lead to public attention directed toward their relatives with mental illness. Therefore, the de-stigmatization of people with mental illness requires more effort in a Chinese context. Education and contact are the main anti-stigma strategies for people with mental illness [32]. Our results showed that the professionals are seeking to create opportunities for interaction with the public through activities such as volunteering or participating in art exhibitions or music performances, to help the public gain a more accurate understanding of people with mental illness. In addition to the efforts being made by individual organizations, education and social interaction require strong support in terms of policies and governmental resources to promote balanced reporting by the media, education on mental health at all levels of education, and to make such information accessible and pervasive throughout society.

Limited policy and resource support are common recovery-oriented service implementation problems [10, 26, 33]. People with mental illness need governmental support to pursue their recovery, yet often having difficulties with attracting attention and support from legislators and governmental officials [34, 35]. For Taiwan, the median mental health expenditure per capita is US$ 0.75 [36], which is lower than the global median mental health expenditure per capita (US$ 2.5) reported by WHO [37]. Current service volume of psychiatric rehabilitation organizations per 10,000 population is 1.45 and 2.88 for day programs and housing programs, respectively [16]. Mental health services in Taiwan are extremely limited, let alone recovery-oriented. It is appreciated that some organizations are willing to embrace the concept of recovery under such difficult conditions. Our results showed that insurance reimbursement for these services needs to be increased. The government can implement mechanisms that enhance organizational motivation for implementing recovery-oriented services, such as extra incentives for individual goal attainment and reimbursements for employment follow up services. In addition, recovery-oriented services include placing value on mutual communication and emphasizing discussions of recovery goals and plans between professionals and people with mental illness, thereby requiring more time and personnel. Governmental policies can increase the number of people allocated in psychiatric rehabilitation organizations to decrease case load and in turn provide quality services. To strive for better services and resources for this population, more effort should be made to promote the effectiveness and advantages of successful recovery-oriented services.

Both the literature and our results reveal the importance of professional education. Even though our participants were from well-established recovery-oriented organizations in Taiwan, they had some colleagues who did not believe in recovery. Education can provide professionals with a more thorough understanding of the concept of recovery and help them establish collaborative relationships with people with mental illness [8, 10, 26]. In addition, the key to successful implementation of recovery-oriented services is to effect changes in the attitudes of the professionals involved [38, 39]. Changes in attitude can be facilitated via training, organization-wide discussions on and support for recovery, as well as courses and case discussions that include professionals sharing their experiences with recovery-oriented services. Training to raise awareness and reflection is also essential in recovery education. Professionals must continually reflect and confirm whether the interaction between themselves and people with mental illness is consistent with the core values of recovery, and it may require a period of practice to find the appropriate pattern. Future recovery education efforts can consider incorporating individual or group reflection activities [40] to help professionals better master the implementation of recovery-oriented services.

The study had several limitations. We interviewed experienced professionals from five well-established recovery-oriented community psychiatric rehabilitation organizations in Taiwan. This may limit the transferability of the findings to those who come from traditional psychiatric rehabilitation organizations, non-community settings, or different cultural backgrounds. Furthermore, social desirability bias may have affected the narratives of the participants who were recommended by the heads of organizations. However, these organizations were recommended by accreditation committee members who are familiar with psychiatric rehabilitation organizations in Taiwan. Few studies have directly discussed the implementation methods used in recovery-oriented services in Asia. This study adds preliminary information contributing to this under-investigated field. In addition, we did not interview other stakeholders, such as policy-makers, family members, and people with mental illness, but rather recruited professionals from various backgrounds, locations, and settings in Taiwan to provide a comprehensive perspective of practitioners and ensure data credibility.

## Conclusions

In this study, mental health professionals who provide recovery-oriented services in Taiwan were interviewed to gain knowledge of their practical experiences. The results documented their experiences with the implementation of recovery-oriented services, the associated problems and strategies used to resolve them, which are beneficial for novices. The influence of Chinese culture on recovery-oriented services was highlighted. Seeking collaboration of family members and combating the effects of stigmatization were found to be the primary challenges faced by professionals in a non-Western context. Policy changes and attitude changes in professionals and people with mental illness were also found to be keys to the success of recovery-oriented services.

## Supplementary Information

**Additional file 1.**

## Data Availability

The datasets generated and/or analyzed during the current study are not publicly available due to the protection of participants’ privacy but are available from the corresponding author on reasonable request.
