# Supplementary material for: Professional perspectives on providing recovery-oriented services in Taiwan: a qualitative study
Source: BMC Psychiatry. 2021 Mar 16;21:154. doi: 10.1186/s12888-021-03152-y (PMC7962288; doi:10.1186/s12888-021-03152-y)
Supplement: Supplementary file 1 — Additional file 1. [file 12888_2021_3152_MOESM1_ESM.docx]

**Title Page**

**Title:**

Professional Perspectives on Providing Recovery-Oriented Services in Taiwan: A Qualitative Study

**Authors and affiliations:**

Yen-Ching Chang^1^, Ling-Hui Chang^1,2^, Su-Ting Hsu^3^, Meng-Wen Huang^4^

^1^Department of Occupational Therapy, College of Medicine, National Cheng Kung University, Tainan, Taiwan

^2^Institute of Allied Health Science, College of Medicine, National Cheng Kung University, Tainan, Taiwan

^3^Kaohsiung Municipal Kai-Syuan Psychiatric Hospital, Kaohsiung, Taiwan

^4^ Department of Rehabilitation Science, Jenteh Junior College of Medicine, Nursing and Management, Miaoli, Taiwan

**The corresponding author:** Meng-Wen Huang

Email: janet19928@hotmail.com

Telephone: +886986619680

Interview Guide

1. How do you implement recovery-oriented services?
   - Please give us some examples.
   - Has your organization formulated relevant policies for recovery?
   - The current accreditation standard of psychiatric rehabilitation organizations includes the concept of recovery. How do you think this will affect the promotion of recovery-oriented services?
2. What challenges do you encounter when implementing recovery-oriented services?
   - Is there anything that needs to be adjusted according to Taiwanese culture?
   - After encountering these challenges, why do you still continue your efforts to implement recovery-oriented services?
3. What strategies do you think can be used to address and resolve these problems?
